# Impacts of fludioxonil resistance on global gene expression in the necrotrophic fungal plant pathogen *Sclerotinia sclerotiorum*

**DOI:** 10.1186/s12864-021-07402-x

**Published:** 2021-01-30

**Authors:** Akeem O. Taiwo, Lincoln A. Harper, Mark C. Derbyshire

**Affiliations:** Centre for Crop and Disease Management, School of Molecular and Life Sciences, Curtin University, Perth, Australia

**Keywords:** Fungicide resistance, *Sclerotinia sclerotiorum*, Fludioxonil, *os1* gene, *Brassica napus*, Histidine kinase

## Abstract

**Background:**

The fungicide fludioxonil over-stimulates the fungal response to osmotic stress, leading to over-accumulation of glycerol and hyphal swelling and bursting. Fludioxonil-resistant fungal strains that are null-mutants for osmotic stress response genes are easily generated through continual sub-culturing on sub-lethal fungicide doses. Using this approach combined with RNA sequencing, we aimed to characterise the effects of mutations in osmotic stress response genes on the transcriptional profile of the important agricultural pathogen *Sclerotinia sclerotiorum* under standard laboratory conditions. Our objective was to understand the impact of disruption of the osmotic stress response on the global transcriptional regulatory network in an important agricultural pathogen.

**Results:**

We generated two fludioxonil-resistant *S. sclerotiorum* strains, which exhibited growth defects and hypersensitivity to osmotic stressors. Both had missense mutations in the homologue of the *Neurospora crassa* osmosensing two component histidine kinase gene *OS1*, and one had a disruptive in-frame deletion in a non-associated gene. RNA sequencing showed that both strains together differentially expressed 269 genes relative to the parent during growth in liquid broth. Of these, 185 (69%) were differentially expressed in both strains in the same direction, indicating similar effects of the different point mutations in *OS1* on the transcriptome. Among these genes were numerous transmembrane transporters and secondary metabolite biosynthetic genes.

**Conclusions:**

Our study is an initial investigation into the kinds of processes regulated through the osmotic stress pathway in *S. sclerotiorum*. It highlights a possible link between secondary metabolism and osmotic stress signalling, which could be followed up in future studies.

**Supplementary Information:**

The online version contains supplementary material available at 10.1186/s12864-021-07402-x.

## Background

Fludioxonil is one of two commercial fungicides, the other being fenpiclonil, derived from the compound pyrrolnitrin, which was first isolated from bacteria in the genus *Pseudomonas* [1, 2]. It is a broad-spectrum fungicide used to control many crop pathogens before and after harvest [3]. It inhibits fungal growth by over-stimulating the high osmolarity glycerol (HOG) pathway to induce hyphal swelling and bursting [4].

The HOG pathway is a branched mitogen activated protein kinase (MAPK) signal transduction system that has been well characterised in *Saccharomyces cerevisiae* [5, 6]. The major role of this pathway is to adapt fungi to the osmolarity of the surrounding environment. Increased osmolarity in the environment leads to water loss and cell shrinkage. To compensate for this, the HOG pathway stimulates production of intracellular glycerol to draw in more water.

A key enzyme in the *S. cerevisiae* HOG pathway is HOG1, which is the final MAPK in the signalling cascade [7]. Phosphorylation of HOG1 leads to transcriptional activation of downstream genes involved in biosynthesis of glycerol. Another key enzyme that may be involved in the HOG response is the *Neurospora crassa* two component histidine kinase known as osmosensing 1 (OS1) [8, 9]. This protein may be involved in the initial response to osmotic stress, prior to activation of the HOG pathway [10].

The reason that fludioxonil is thought to over-stimulate the HOG pathway is that null mutants for genes in this pathway are often resistant to it, as well as being hypersensitive to osmotic stress [11]. Furthermore, exposure to fludioxonil seems to stimulate production of glycerol under normal growth conditions, analogous to the addition of osmolytes such as sorbitol or NaCl_2_ to the growth medium [12].

Although there are few instances of fludioxonil resistance among field isolates of pathogens [3], laboratory mutants are easily induced through continual exposure to sub-lethal doses of the fungicide [13–17]. Most of these mutants harbour mutations in members of the HOG pathway or related genes such as *OS1*. Presumably, these mutations inactivate the osmotic stress response, leading to a phenotype that is analogous to the null mutants developed through targeted gene deletions or other means.

Laboratory mutants and targeted null mutants alike often have marked physiological and growth defects. For example, in *Parastagonospora nodorum*, deletion of *HOG1* led to reduced production of pycnidia. Furthermore, *P. nodorum ∆hog1* strains were not only susceptible to osmotic stress in vitro but heat stress as well [18]. In *B. cinerea*, laboratory mutants resistant to fludioxonil that had mutations in the homologue of *N. crassa OS1* were less virulent on strawberry and tomato leaves and grew more slowly in vitro [14]. Defects such as these could be caused by the multifaceted role of the HOG pathway, which is known to be involved in response to a variety of stressors other than high osmolarity [19–24].

Some responses to cellular stress, such as accumulation of glycerol under hyperosmotic conditions, have an obvious compensatory function. However, others may have more cryptic roles in nature. For example, fungal secondary metabolites are compounds that are, by definition, unnecessary for normal growth under non-stress conditions. These compounds have various roles in nature such as microbial competition, defence against parasites and mitigation of the harmful effects of environmental stressors such as ultraviolet light [25]. MAPK cascades such as the HOG pathway may mediate their production in response to stress. For example, in *Fusarium graminearum*, individual null mutants for the three MAPKs of the HOG pathway showed enhanced pigmentation and over-expression of the aurofusarin biosynthetic pathway and reduction of trichothecene production [26].

The fungus *S. sclerotiorum* is a broad host range pathogen that infects hundreds of plant species [27]. It is controlled on some crops using fludioxonil to which it shows no evidence of resistance in the field. Laboratory mutants of *S. sclerotiorum* resistant to fludioxonil have shown physiological defects similar to those in other fungi, including reduced growth and pathogenicity [15]. However, little is known about the broader transcriptional impact of mutations in osmotic stress response genes in *S. sclerotiorum* or other fungi. Therefore, we generated two fludioxonil-resistant *S. sclerotiorum* laboratory strains that were confirmed to have independent mutations in the *S. sclerotiorum* homologue of *N. crassa OS1* and sequenced their transcriptomes under standard in vitro culture conditions.

Both of these strains exhibited a similar transcriptional profile that was different to their parent. Changes in expression of some genes downstream of the HOG pathway in yeast was observed. In addition, many secondary metabolite biosynthetic clusters, including those involved in carotenoid biosynthesis, were affected in both of these mutants. This highlights a potential link between secondary metabolism and HOG signalling in *S. sclerotiorum*.

## Results

### Fludioxonil-resistant strains of *Sclerotinia sclerotiorum* have defects in in vitro growth and pathogenicity

**Fig. 1:**
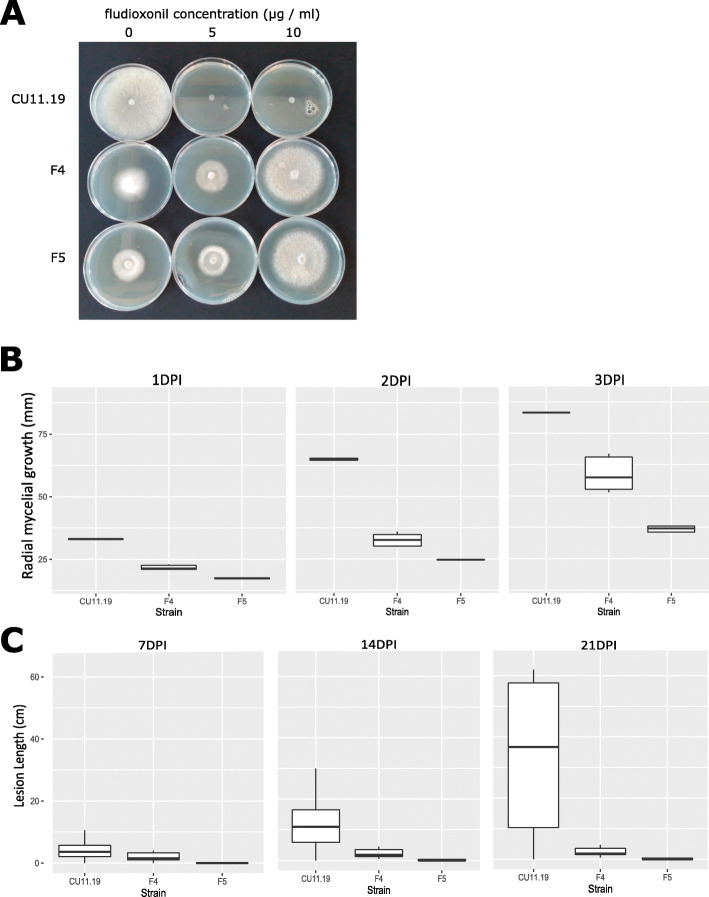
Sensitivity to fludioxonil and growth defects in *Sclerotinia sclerotiorum* mutant strains. **a** The image shows representative plates of the two fludioxonil-resistant and the wild-type isolate CU11.19 strains after 3 days of growth on potato dextrose agar (PDA) amended with 5 and 10 μg / ml fludioxonil. No growth was observed in the wild-type parent strain CU11.19 at this fungicide concentration. **b** The y axis shows radial growth (mm) after 1, 2 and 3 days post-inoculation (1DPI, 2DPI and 3 DPI) of PDA for each of the strains, CU11.19, and F4 and F5. Thick horizontal lines show medians and boxes and whiskers show interquartile range. **c** The same format as for **b** but showing lesion length on *Brassica napus* stems at seven, 14 and 21 DPI

We generated fludioxonil-resistant strains of *S. sclerotiorum* by continual sub-culturing on inhibitory doses of the fungicide. To assess their levels of resistance to fludioxonil, a discriminatory dose assay comparing them with their parent was performed. On the control plates, which had no fludioxonil added, mycelial growth was observed for the parent CU11.19, while no growth was observed at 5 μg / ml and 10 μg / ml fludioxonil. The two fludioxonil-resistant strains F4 and F5 exhibited mycelial growth on the control plates and on both fungicide concentrations (Fig. 1a). This suggests that F4 and F5 were resistant to fludioxonil.

Fludioxonil-resistant strains of numerous fungi exhibit growth defects. Therefore, we assessed growth rates of F4 and F5 in vitro. We found that the parent strain, CU11.19, had the highest mean radial mycelial growth at each time point. Despite the fact that F4 and F5 also grew on the control plates, their mycelial growth rates were significantly lower (*P* = 0.0024, *n* = 15, α = 0.05) than that observed for the WT (Fig. 1b), which underscores the significant growth defects observed in the fludioxonil strains.

We also assessed pathogenicity of these strains on the *S. sclerotiorum* host species *B. napus*. We found that the parent strain, CU11.19, produced the highest mean lesion lengths (4.47, 11.0 and 32.79 cm) at seven, 14 and 21 days post-inoculation (DPI). These lengths were significantly greater (*P* = 0.002, *n* = 30, α = 0.05) than those observed for F4 (1.93, 2.21 and 2.33) and F5 (0.2, 0.46 and 0.5) (Fig. 1c). This shows that both fludioxonil-resistant strains were less pathogenic than the parent strain.

### Fludioxonil-resistant strains of *Sclerotinia sclerotiorum* are less tolerant of osmotic stress conditions in vitro

**Fig. 2:**
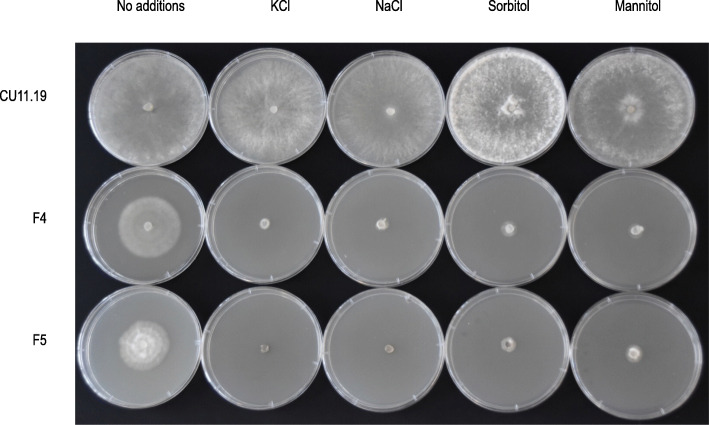
Sensitivity to hyperosmotic stress in the fludioxonil-resistant strains. From left to right shows growth after 4 days on potato dextrose agar (PDA) with no amendments, with 0.5 M KCl, NaCl, Sorbitol and Mannitol. The top row is the wild-type parent strain CU11.19 and rows two and three are the mutant strains F4 and F5, respectively. Whereas CU11.19 grew on medium containing the osmolytes, the fludioxonil-resistant strains did not

Fungal mutants, including *∆os1* strains, that have defects in HOG signalling are often hypersensitive to osmotic stress [8]. Therefore, we grew the two mutant *S. sclerotiorum* strains under hyperosmotic conditions to test their sensitivity to osmotic stress. We found that both F4 and F5 did not exhibit any growth on potato dextrose agar medium containing the salts KCl and NaCl, whereas their wild-type parent did. Similarly, F4 and F5 did not exhibit any growth on medium containing the sugars sorbitol and mannitol, whereas the wild-type parent did (Fig. 2). Overall, this shows that the fludioxonil-resistant *S. sclerotiorum* strains were hypersensitive to osmotic stress.

### Fludioxonil-resistant strains exhibit a handful of genome-wide mutations and both have a modified OS1 sequence

The objective of our study was to assess the impacts of mutations in osmotic stress response pathway genes on the broader transcriptome. So far, we have shown that the fludioxonil-resistant strains were less tolerant of osmotic stress but we wished to determine the likely mutations that were responsible for this phenotype. To do this, we performed whole genome sequencing.

A total of 11 mutations were found in association with six genes in F5, while four mutations associated with one gene were found in F4 (Table 1). Most of these mutations were intergenic, occurring fewer than 5 Kb from the nearest coding sequence. However, in F5, the gene sscle_10g077450 harboured a disruptive in-frame deletion, and in both F4 and F5 the gene sscle_02g013550 harboured a missense mutation. The latter gene is homologous to *OS1* from *N. crassa* (amino acid identity = 79%), forming a one-to-one orthologous group in the OrthoFinder output.

**Table 1:** Description of mutations identified in mutants F4 and F5 from comparative genomic analyses with parent CU11.19

Gene ID	Strain	Mutation impact on gene	Mutation type	Nucleotide Change
sscle_02g013550	F5	missense_variant MODERATE	Missense	C > T
sscle_04g033580	F5	upstream_gene_variant MODIFIER	Intergenic	C > T
sscle_05g043100	F5	downstream_gene_variant MODIFIER	Intergenic	685 bp deletion
sscle_10g077450	F5	disruptive_inframe_deletion	Frameshift	157 bp deletion
sscle_11g082170	F5	upstream_gene_variant MODIFIER	Intergenic	C > T
sscle_11g082170	F5	upstream_gene_variant MODIFIER	Intergenic	A > G
sscle_11g082170	F5	upstream_gene_variant MODIFIER	Intergenic	T > C
sscle_11g082170	F5	upstream_gene_variant MODIFIER	Intergenic	C > A
sscle_11g084380	F5	upstream_gene_variant MODIFIER	Intergenic	T > C
sscle_11g084380	F5	upstream_gene_variant MODIFIER	Intergenic	T > TC
sscle_11g084380	F5	upstream_gene_variant MODIFIER	Intergenic	T > C
sscle_02g013550	F4	missense_variant MODERATE	Missense	C > T
sscle_04g034270	F4	upstream_gene_variant MODIFIER	Intergenic	G > GT
sscle_05g046610	F4	upstream_gene_variant MODIFIER	Intergenic	A > G
sscle_11g084380	F4 & F5	upstream_gene_variant MODIFIER	Intergenic	G > A

**Fig. 3:**
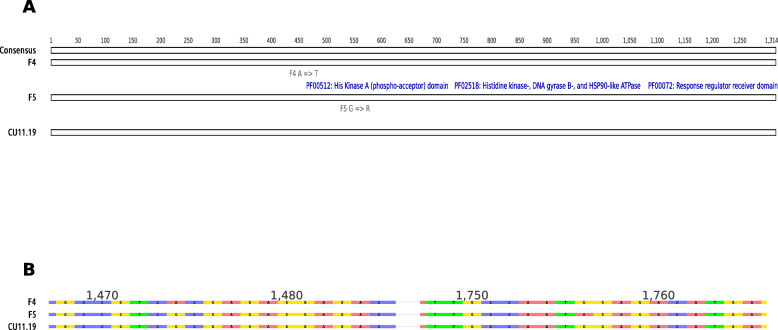
Mutations in the *Sclerotinia sclerotiorum os1* gene likely conferred resistance to fludioxonil. **a** Alignment of the OS1 protein sequences of the strains F4 and F5 and their parent strain CU11.19. The top line is the consensus sequence. Each amino acid is represented in a different colour. The blue bars represent PFAM domains present in the OS1 protein. The grey bars represent the amino acid substitutions present in F4 and F5. **b** The non-synonymous nucleotide substitutions in F4 and F5 that caused missense amino acid replacements in the OS1 protein. A point mutation at position 1474 of the *OS1* coding DNA sequence (CDS) from G to A in F4 caused a non-synonymous mutation from Alanine (A) to Threonine (T). A point mutation at position 1756 of the *OS1* CDS from G to A in F5 caused a non-synonymous mutation from Glycine (G) to Arginine (R)

There was a different missense mutation in this gene in the two different strains. These mutations both occurred between the HAMP (found in Histidine kinases, Adenylate cyclases, Methyl accepting proteins and Phosphatases) and His kinase A (phosphor-acceptor) domains [28] (Fig. 3a). In F4, a non-synonymous mutation at position 1474 from G to A resulted in an amino acid substitution from Alanine (A) to Threonine (T). In F5, a non-synonymous mutation at position 1756 from G to A caused an amino acid substitution from Glycine (G) to Arginine (R) (Fig. 3b). Given the known role in fludioxonil resistance of the HOG pathway and the fact that this was the only gene that exhibited mutations in both strains, it is likely that this was the basis of the resistance phenotype observed in F4 and F5.

### The two fludioxonil-resistant strains exhibited similar transcriptomic profiles that were distinct from their parent

**Fig. 4:**
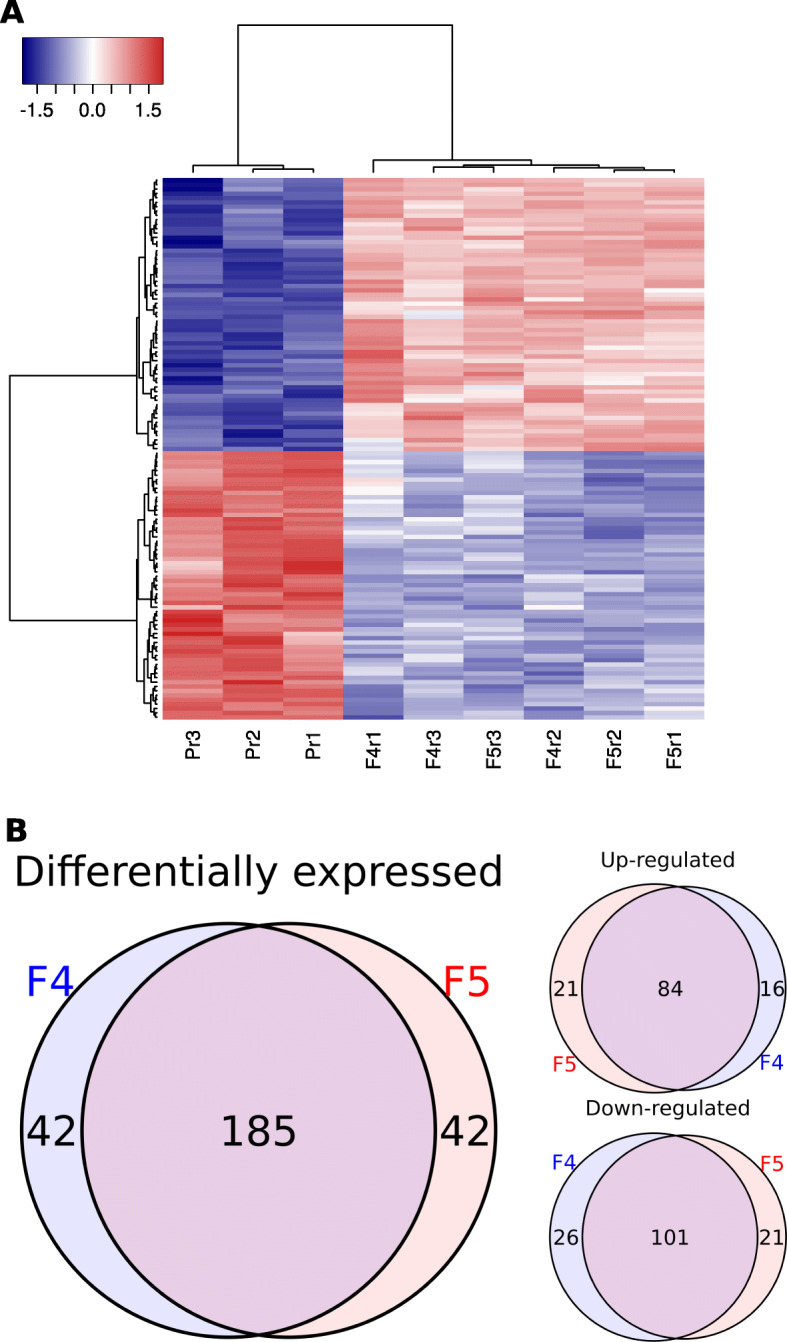
The two fludioxonil-resistant strains exhibited similar transcriptional profiles during in vitro growth. **a** The top 100 differentially expressed genes, based on ranking of *P* values, across both strains. Hierarchical clustering grouped the two strains together, away from the wild-type parent (CU11.19). The colouring represents log_2_(edgeR normalised expression), going from blue (low) to red (high). **b** Venn diagrams showing the number of genes differentially expressed across F4 and F5 relative to the parent strain. The intersection consists of genes that were differentially expressed in both strains in the same direction relative to expression in CU11.19

Given the observation that both F4 and F5 harboured a non-synonymous mutation in an *S. sclerotiorum* homologue of a HOG pathway-related gene, we speculated that they may have similar perturbations in the HOG pathway. If this were the case, we would expect to observe similar alterations in the transcriptome relative to the fungicide-sensitive parent CU11.19. We found that 269 genes were differentially expressed across both strains relative to CU11.19. Out of these, 121 were upregulated and 148 were down regulated. Of the genes unique in their differential expression to F4, 16 were up and 26 downregulated. In F5, 21 genes were uniquely upregulated and 21 uniquely downregulated (Fig. 4a and b). However, 185 (69%) genes were differentially expressed in both F4 and F5 in the same direction, with 84 up-regulated and 101 down-regulated. The two strains also clustered together, away from the parent, in a hierarchical clustering analysis based on their gene expression profiles (Fig. 4a is a heatmap based on the top 100 *P* values and Supplementary Figure 1 is a heatmap based on the top 500 *P* values). Since the transcriptomes of these two independent strains were so similar, it is likely that they suffered similar physiological impacts from their different missense mutations in the *OS1* homologue. By investigating the functional domains of the differentially expressed genes, we thus hoped to uncover some aspects of OS1 and HOG pathway regulation in *S. sclerotiorum*.

### Enzymes transcriptionally regulated by members of the HOG pathway show alterations in expression in both fludioxonil-resistant strains

**Fig. 5:**
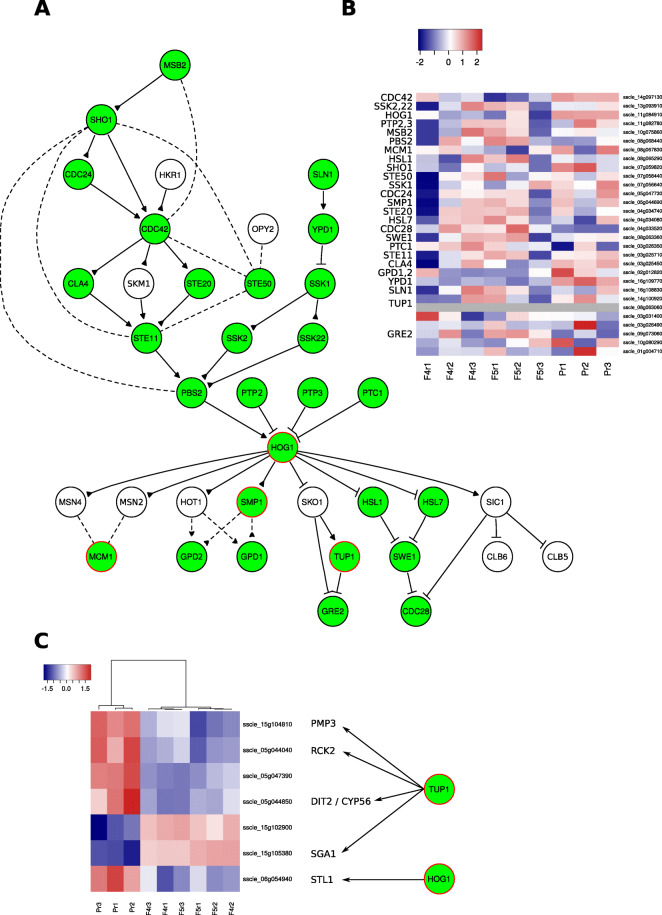
Expression of genes associated with the high osmolarity glycerol pathway in the fludioxonil-resistant strains. **a** A reproduction of the high osmolarity glycerol (HOG) pathway of *Saccharomyces cerevisiae*. Green circles had homologues in the *Sclerotinia sclerotiorum* proteome. Those outlined in red are known to be involved in transcriptional regulation of other genes. **b** Expression of the HOG pathway homologues in *S. sclerotiorum*. Again, expression is log_2_(edgeR normalised expression) going from low (blue) to high (red). None of the *S. sclerotiorum* genes homologous to yeast HOG pathway genes were significantly differentially expressed in either F4 or F5. **c** Expression of genes downstream of transcriptional regulators from the HOG pathway. Seven genes in *S. sclerotiorum* were differentially expressed in F4 and F5 and were homologous to *S. cerevisiae* genes known to be regulated by HOG pathway associated genes. These genes are known to be regulated by HOG1 and TUP1

An obvious place to start elucidating the role of the HOG pathway in gene expression regulation in these mutant strains is the well-characterised genes of *S. cerevisiae*. Using the protein sequences encoded by these genes, we searched for homologues in the *S. sclerotiorum* proteome. We found that many of the yeast HOG pathway genes had homologues in *S. sclerotiorum*, including all three MAPKs, STE11 (the MAPKKK), PBS2 (the MAPKK) and HOG1 (the MAPK) (Fig. 5a). Most of these genes were one-to-one homologues. However, the genes TUP1 and GRE2 were present in two and five copies, respectively, in *S. sclerotiorum*.

None of the *S. sclerotiorum* HOG pathway homologues were differentially expressed in either F4 or F5 (Fig. 5b). However, using the *S. cerevisiae* genome portal we were able to identify genes transcriptionally regulated by HOG1, SMP1, MCM1 and TUP1 that were not actually members of the pathway themselves. Some homologues of genes transcriptionally regulated by TUP1 and HOG1 in *S. sclerotiorum* showed evidence of differential expression in both fludioxonil-resistant strains (Fig. 5c). Homologues of the TUP1-regulated genes PMP3 and RCK2 were downregulated in both strains. The TUP1-regulated gene DIT2 / CYP56 was present in three copies in *S. sclerotiorum*, two of which were downregulated in both strains and one up-regulated. The HOG1-regulated gene STL1 was significantly downregulated in both strains. Overall, our data suggest that there was no impact directly on expression of HOG pathway genes in *S. sclerotiorum* in the fludioxonil-resistant strains. However, there was some evidence of altered expression of non-HOG pathway genes transcriptionally regulated by a few HOG pathway members.

### Transmembrane transporter genes are over-represented in the differentially expressed set shared between the fludioxonil-resistant strains of *Sclerotinia sclerotiorum*

**Fig. 6:**
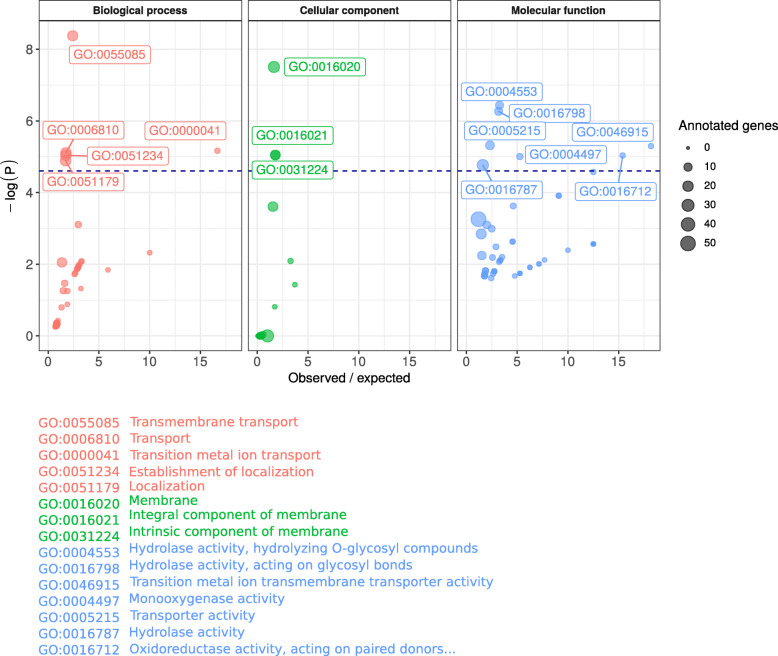
Gene Ontology term enrichment analysis of the 185 genes differentially expressed in both mutant strains. The y axis shows -log(P) of the Gene Ontology (GO) term enrichment test after false discovery rate correction. The size of points represents the number of genes annotated with the different GO terms. The x axis represents the number of genes with that GO term observed over the number expected given the frequency at which that GO term was observed in the rest of the genome

Having established that the two strains had independent mutations in *OS1* and exhibited broadly similar transcriptomes, we aimed to determine what kinds of biological process were impacted at the transcriptomic level in response to these mutations. To do this, we performed a GO term enrichment analysis on the gene set that was differentially expressed in both strains relative to the parent. We found that several GO terms were over-represented among these 185 genes. In particular, terms associated with membrane transport such as ‘Transmembrane transport’ (GO:0055085), ‘Transition metal ion transport’ (GO:0000041), ‘Membrane’ (GO:0016020) and ‘Transition metal ion transmembrane transporter activity’ (GO:0046915) (Fig. 6, Supplementary Table 1).

### Many secondary metabolite biosynthetic clusters have altered expression in the *Sclerotinia sclerotiorum* fludioxonil-resistant strains

There are known links between secondary metabolism and response to cellular stresses in fungi. In addition to our broader assessments of the transcriptome, we aimed to specifically characterise the impacts of mutations in *OS1* on expression of secondary metabolite clusters. To do this, we used the manually curated secondary metabolite biosynthetic clusters identified by Graham-Taylor et al. (2020) [29].

**Fig. 7:**
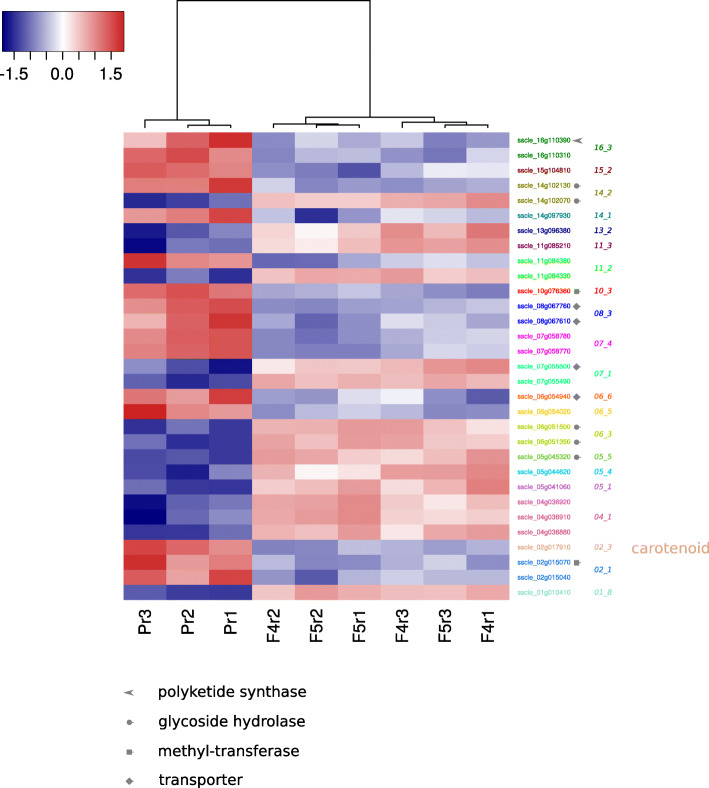
Differential expression of secondary metabolite biosynthetic genes in the fludioxonil-resistant strains. Expression is log_2_(edgeR normalised expression), which goes from low (blue) to high (red). All genes that were differentially expressed in the same direction in both strains and that also occurred in secondary metabolite biosynthetic clusters are given to the right. Those of the same colour are in the same secondary metabolite biosynthetic clusters and their cluster IDs are also given. The different symbols refer to the different functional domains present in these genes (listed below)

We identified 31 genes present in these secondary metabolite biosynthetic clusters that were differentially expressed in the same direction in both F4 and F5 (Fig. 7). A total of 16 of these genes were downregulated and the other 15 upregulated. Among these genes, one was a polyketide synthase, five were glycoside hydrolases, two were methyl-transferases and four were transporters. One of the genes, ‘sscle_02g017910’, is a taurine catabolism dioxygenase (PF02668) that is part of a cluster homologous to carotenoid biosynthetic clusters in other fungi. This gene was downregulated in the two fludioxonil-resistant strains. Overall, these data suggest that common osmosensing perturbations in these two strains led to differential regulation of secondary metabolite biosynthetic genes, including those involved in carotenoid biosynthetic.

## Discussion

In our study, we generated two fludioxonil-resistant strains of *S. sclerotiorum*. Both strains had missense mutations in the *S. sclerotiorum* homologue of *N. crassa* OS1. Disruption of the osmotic stress response in our strains was supported by the observation that they were less tolerant of hyperosmotic conditions than the parent strain.

In addition, the two strains had marked growth defects, which is similar to observations in other fungi. For example, *B. cinerea* fludioxonil-resistant strains developed through laboratory selection had different non-synonymous mutations in *OS1*. These isolates had reduced rates of growth and significant reductions - up to 100% in some cases - in sporulation [14]. In *N. crassa*, deletion of *OS1* results in aberrant hyphal morphology; aerial hyphae in these strains are bulbous and prone to lysing [9].

Any mutations that inactivate *os1* are likely to be both non-lethal and to induce fludioxonil-resistance. This makes fludioxonil-resistant strains easy to generate in the lab through experimental evolution. However, the extensive physiological defects of *os1* null mutants in most fungi likely precludes their survival in the field [3]. For example, despite the widespread use of fludioxonil for preventing grey mold, *B. cinerea os1* null mutants with fludioxonil-resistance have only been sampled once at very low frequency from the field [14]. Other mechanisms conferring partial resistance to fludioxonil may be more relevant to industry. For example, several *B. cinerea* field isolates harbour so-called multi-drug resistance [30]. These strains overexpress efflux transporter genes that encode proteins capable of forcing multiple fungicide compounds, including fludioxonil, out of the cell [31]. There is thus an important distinction between *os1* null mutants generated in the laboratory and field isolates with fludioxonil-resistance.

The main purpose of our study was to assess the impacts of mutations in *OS1* in global transcriptional regulatory networks. Although we cannot say for sure, we infer that the two mutant strains likely exhibited aberrant *OS1* activity. This is because the only gene that harboured mutations in both strains was *OS1* and the two strains had defects typical of null mutants for osmotic stress response genes.

The independent mutations in *OS1* in the different strains likely had similar impacts on the transcriptome. The 269 genes impacted in expression in these strains highlights the multifaceted role of the HOG pathway in direct and indirect transcriptional regulation. It also indicates that the two strains, despite their different *OS1* missense mutations, likely had similar changes in their gene regulatory networks.

To assess the direct impacts of the OS1 mutations on osmotic stress signalling, we investigated the expression of homologues of the *S. cerevisiae* HOG pathway in *S. sclerotiorum*. However, we found that none of these genes were differentially expressed in the mutant strains. This fits with the general signal transduction mechanism, which involves protein-protein interactions rather than transcriptional activation or repression.

Looking outside of the HOG pathway, we then aimed to assess the impacts on expression of genes that are known to be transcriptionally regulated by pathway members in yeast. We found that homologues of some of these genes were indeed differentially expressed in the mutant *S. sclerotiorum* strains. Three were paralogues in *S. sclerotiorum* that were homologous to the *S. cerevisiae* gene *DIT2*. This gene is usually expressed in the middle phase of sporulation, and it encodes an enzyme that is involved in biosynthesis of dityrosine –a compound incorporated into the outermost layer of the spore wall [32]. During vegetative growth, *DIT2* expression is repressed by a SSN6-TUP1 complex [33]. Two of the *S. sclerotiorum DIT2* homologues were downregulated in the mutant strains, whereas one was upregulated. Though we do not know the precise functions of these genes, we can speculate that they respond to the external environment via *OS1* in *S. sclerotiorum*. Thus, *OS1* may be involved in a developmental process in *S. sclerotiorum* that is analogous to sporulation in yeast.

We also investigated the expression of secondary metabolite biosynthetic cluster genes in these strains, as secondary metabolism is often linked with response to environmental stress in fungi. We found that a number of genes in putative secondary metabolite biosynthetic clusters were differentially expressed in both fludioxonil-resistant strains. This has some precedent from other studies. For example, a *Fusarium graminearum ∆os1* strain produced less of the red secondary metabolite pigment aurofusarin [26]. Interestingly, deletion mutants for other members of the HOG pathway, including *OS4*, *OS2* and *OS5*, showed enhanced pigmentation and reduction of the mycotoxin deoxynivalenol (DON). This is possibly suggestive of differing secondary metabolism regulatory roles of different points in the HOG pathway. Furthermore, another study in *F. graminearum* showed that the gene *Sch9* is an integral mediator of the transcriptional response induced by the nutrient sensing (target of rapamycin (TOR)) and HOG pathways. Interestingly, this protein co-precipitates with both HOG1 itself and the core component of the TOR pathway. Deletion of this gene in *F. graminearum* caused a reduction in production of DON [34]. Although the exact links between osmosensing and secondary metabolism are fairly elusive in fungi, we can speculate that *OS1* in *S. sclerotiorum* is somehow involved in both secondary metabolite suppression and activation during in vitro growth.

A major limitation of the current study is that we did not use targeted gene deletion strains for *OS1*. This was a particular issue with the strain F5, which had a major disruptive mutation in a non-target gene. However, given the weight of literature supporting defects in osmosensing in fludioxonil-resistant strains and the known role of OS1 in this pathway, we infer that our observations are truly reflective of perturbations in this pathway and not mutations in other parts of the genome. The similarity in transcriptomic response of both strains supports this inference. Future research could include targeted gene deletion of *OS1* in *S. sclerotiorum*, which may elucidate the effects of the non-*OS1* mutation we observed in F5.

Another limitation of the current study is that we sequenced the transcriptomes of the two strains under standard laboratory conditions, without osmotic stress exposure. This only allows us to determine differences in basal gene expression between the parent and the mutant strains. Although it gives us some insight into the kinds of genes regulated by OS1 and the HOG pathway, genes that would have changed expression in response to osmotic stress cannot be identified. Future studies could add to this RNA sequencing data set, using more data generated under osmotic stress conditions.

## Conclusions

In conclusion, we generated two laboratory mutants resistant to fludioxonil by continual subculturing on sublethal doses of the fungicide with the purpose of understanding the transcriptional impacts of mutations in osmosensing genes. These two strains were likely resistant to fludioxonil because of mutations in *OS1* and perturbations in the osmosensing signalling cascade. The physiological and growth defects observed in these strains were reflected in a substantially altered transcriptome under standard in vitro conditions. Several genes homologous to those transcriptionally regulated by HOG pathway members were differentially expressed in these strains. These included three genes homologous to the yeast spore wall associated gene *DIT2*, one of which was down-regulated and two up-regulated in the mutant strains. This suggests that *OS1* is in some way involved in an analogous process in *S. sclerotiorum*. In addition, a number of genes from previously identified secondary metabolite biosynthetic clusters were differentially expressed in the fludioxonil-resistant strains. This highlights a link between osmosensing and secondary metabolite production in *S. sclerotiorum.* Under basal conditions, it is likely that *OS1* has a role in the signalling cascades that lead to secondary metabolite production in this species.

## Methods

### Culturing of *Sclerotinia sclerotiorum* and media preparation

The *S. sclerotiorum* isolate CU11.19 was selected based on the history of its pathogenicity on canola in Australia [35]. CU11.19 was cultured by sterilising sclerotia in 70% ethanol, rinsing them with sterile water, then cutting them into two halves and placing them on Potato Dextrose Agar (PDA) plates facing down under a laminar flow hood to prevent contamination. The plates were incubated at 20 °C in the dark for 4 days. The potato dextrose agar (PDA) medium was prepared by dissolving 39 g of PDA (200 g of potato, 20 g of agar and 20 g of dextrose) in one litre of distilled water. The mixture was autoclaved for 2 h before pouring onto plates.

### Generation of fludioxonil-resistant *Sclerotinia sclerotiorum* strains from in vitro cultures

To select for fludioxonil-resistant laboratory strains of *S. sclerotiorum*, 40 mycelial plugs of *S. sclerotiorum* were obtained from the edge of an actively growing colony on PDA. The plugs were placed in a grid like pattern on 90 mm PDA plates, amended with 1 μg / ml of fludioxonil. The plates were incubated at 20 °C in the dark for 3 weeks. Plugs exhibiting fast-growing mycelium were sub-cultured, with each placed on freshly prepared PDA amended with 1 μg / ml of fludioxonil. This selection process was repeated on further plates until at least two mutants were found. A total of six fludioxonil-resistant strains were isolated. Two of these were chosen for further experimentation and they are referred to as strains ‘F4’ and ‘F5’. The sclerotia and mycelium on agar plugs obtained from the laboratory resistant strains of *S. sclerotiorum* were stored at 4 °C.

### Assessment of growth of CU11.19 and fludioxonil-resistant laboratory cultures on discriminatory concentrations of fludioxonil

Mycelial plugs of resistant and parent strains of *S. sclerotiorum* obtained from freshly prepared culture were placed on PDA amended with fludioxonil (0, 5 and 10 μg / ml). Each concentration was replicated five times and plates were incubated at 20 °C for 7 days. Mycelial growth of the resistant strains and the parent was observed under each concentration and scored as positive or negative. ‘Positive’ refers to gradual spread of the mycelium across the plate, whereas ‘negative’ refers to no growth at all.

### Assessment of the growth rates of the parent and fludioxonil-resistant laboratory strains

Agar plugs containing mycelium of resistant or parent strains of *S. sclerotiorum* obtained from freshly prepared culture were inoculated onto PDA plates without fungicide. Each plate was replicated five times. The plates were incubated at 20 °C. Radial growth of the mycelium of each strain was measured with a Vernier Caliper in two perpendicular directions at one, two and three DPI.

### Assessment of sensitivity of mutant strains to hyperosmotic stress

The sensitivity of the resistant strains to hyperosmotic stress was tested on two salt compounds (NaCl and KCl) and two sugar compounds (sorbitol and mannitol). Agar plugs containing mycelium of resistant or parent strains of *S. sclerotiorum* were inoculated onto freshly prepared PDA plates containing a 0.5 M concentration of each compound prepared separately. Each plate was replicated ten times and incubated at 20 °C. Mycelial growth of the resistant strains and the parent was observed under each compound after four DPI.

### Assessment of pathogenicity of the parent and fludioxonil-resistant laboratory strains

A pot experiment was carried out to determine the pathogenicity of the fludioxonil-resistant strains of *S. sclerotiorum* on *B. napus* plants. The experiment comprised of two fludioxonil-resistant laboratory strains (F4 and F5) and the parent (CU11.19) of *S. sclerotiorum*. The experiment was carried out on the *B. napus* cultivar Tribune, which has been commercially grown in Australia, where *S. sclerotiorum* is a significant issue, and is not known to be resistant. *B. napus* seeds were sown in the pot (4 seeds / pot) and thinned to one plant per pot after 4 weeks of growth. The plants were grown in a plant growth chamber with a 12-h photoperiod and 22 °C temperature cycle. The relative humidity was set at 60% and day light intensity was 130 μmol / m^2^ / s. At 50% flowering, each plant was inoculated with a PDA plug containing mycelium obtained from F4, F5 or CU11.19 plate cultures. These cultures were grown from sclerotia derived from previous plate cultures stored at 4 °C. The plugs were bound to the stem with Parafilm®, with the mycelial plug in contact with the stem. The cultures were prepared from their respective sclerotia as described above. Lesion lengths on stems were measured at seven, 14 and 21 DPI. The experiment had a randomised design with ten replicates.

### Extraction of DNA and Illumina sequencing

The fludioxonil-resistant strains F4 and F5 and parent strain CU11.19 of *S. sclerotiorum* were cultured on PDA as described above. Four plugs of actively growing mycelium were used to inoculate 100 ml of potato dextrose broth (PDB) in an Erlenmeyer flask and grown for 3 days at 150 RPM at room temperature. Fungal tissue was washed in sterile distilled water, snap-frozen in liquid nitrogen and freeze-dried before DNA extraction. The DNA was extracted using the procedure described in [36]. DNA sequencing was conducted by Novogene (Wan Chai, Hong Kong, China) using an Illumina® (San Diego, CA, USA) based method to obtain one gigabase (Gb) of 150 base pair (bp) paired-end reads. The library preparation was carried out with the NEBNext® Ultra™ Library Prep Kit for Illumina® and sequencing was performed on an Illumina NovaSeq PE150.

### Extraction of RNA and RNA sequencing

Mycelium of the parent and fludioxonil-resistant laboratory strains were obtained as described above. Both fludioxonil-resistant strains and WT strains were grown at the same time for the same amount of time (3 days) under the same conditions (shaking at 150 RPM at room temperature) in PDB, then mycelium was collected at the same time. RNA was extracted from samples using the TRIzol method (Invitro Corp., Carlsbad, CA, USA). RNA concentration and quality were assessed using a nanodrop and gel electrophoresis. The samples were stored at − 80 °C. RNA sequencing was conducted by Novogene (Wan Chai, Hong Kong, China) using an Illumina® (San Diego, CA, USA) based method to generate 20 million 150 bp paired-end reads per sample. The sequencing library was prepared with the NEBNext® Ultra™ RNA Library Prep Kit for Illumina® and sequencing was performed on the NovaSeq PE150.

### Statistical analyses

Data generated from the fitness assessment and pathogenicity assessments were subjected to analysis of variance (ANOVA) using the R statistical package [37] version 4.0.3. Data were screened for normality and homogeneity of variance and histograms of residuals from the two models showing an approximately normal distribution are in Supplementary Figure 2. Mean differences were compared using Tukey’s honest significant difference (HSD) post hoc test. All plots were generated using the R package ‘ggplot2’ [36].

### Whole genome sequence bioinformatic analysis

Fragments of Illumina adapters and poor-quality sequence were removed from reads using the software package Trimmomatic version 0.36 [38], with the settings ILLUMINACLIP:Truseq3 adapters.fasta:1:30:12 LEADING:20 TRAILING:20 SLIDINGWINDOW:4:5′, where’Truse3 adapters.fasta’ is a file containing adapters from the Illumina Truseq3 library prep kit. Reads of the isolates CU11.19, F4 and F5 and the chromosomes of the complete genome of *S. sclerotiorum* isolate 1980 [39] were compared using the coloured the Bruijn graph method implemented in McCortex (version 1.0.1, release date 05/27/2018) [40]. Only variants that had a GQ value of more than five that differed between CU11.19 and F4 or F5 were kept. The program SnpEff version 4.3 t [41] was used to determine effects of variant on genes using the high-quality annotations of the *S. sclerotiorum* reference genome. The likely effects of SNPs were annotated with respect to the reference genes of *S. sclerotiorum*. Where a reference gene ID prefixed with ‘sscle_’ is discussed as having a certain kind of mutation in one of the re-sequenced strains, we are referring to SNPs called in these strains that likely had an impact on that gene.

### Detection of *Sclerotinia sclerotiorum* homologues of previously characterised genes

The homologue of the OS1 protein from *N. crassa* (GenBank accession: XP_964471.1) was identified in the *S. sclerotiorum* reference proteome as the protein ‘sscle_02g013550’ (GenBank accession APA06585.1) using OrthoFinder version 2.4.0 [42]. The inputs for OrthoFinder were the *S. sclerotiorum* strain 1980 reference proteome (GenBank BioProject ID: PRJNA348385) and the genome of *N. crassa strain* ATCC 24698 downloaded from EnsemblFungi [43]. OrthoFinder was also used to infer orthology relationships between the proteins of *S. cerevisiae* (downloaded from the *Saccharomyces* genome database [44]) and *S. sclerotiorum* to interrogate potential perturbations in genes linked to the HOG pathway. In both instances, we did not assess gene duplication events or relationships between paralogues as we were only interested in broadly describing the relationships between the genes. For instance, where multiple recent paralogues were found in *S. sclerotiorum* that had more similarity to each other than they did to their yeast homologue, we did not investigate their evolutionary history in a broader taxonomic sample. Further, we did not investigate cryptic orthology relationships where a gene may have been duplicated and its original copy lost as this is not in the scope of the current study.

The sscle_02g013550 protein sequence from the reference strain was then aligned to the assembled genomes of F4, F5 and CU11.19 using Exonerate version 2.4.0 [45] with the options ‘--model est2genome’ and ‘--bestn 1’. The gene sequences of *os1* were extracted from the assembled genomes based on the coordinates of the alignment of sscle_02g013550 using the Bedtools version 2.26.0 [46] tool ‘getfasta’. The CDS sequences of these were identified via MUSCLE [47] (in Geneious version 9.0.5) alignment with the CDS sequence of sscle_02g013550 downloaded from GenBank. Concatenated CDS sequences and their translations were then aligned using MUSCLE (in Geneious version 9.0.5).

### Differential expression analysis

Paired reads from F4, F5 and CU11.19 (in three replicates) were aligned to the *S. sclerotiorum* strain 1980 reference genome using Bowtie 2 version 2.3.4.1 [48] with default settings. The program HT-seq count version 0.12.4 was then used alongside the reference genome gene annotations to generate a table of counts for each gene. The count table was used as input for edgeR version 3.26.8 [49]. The quasi-likelihood ratio test of differential expression was used to assess differential expression between either CU11.19 and F4 or CU11.19 and F5. *P* values were adjusted using the method of Benjamini and Hochberg [50] and genes with an adjusted *P* value below 0.01 were designated as differentially expressed.

## Supplementary Information

**Additional file 1: Figure S1.**

**Additional file 2: Table S1.**

**Additional file 3: Figure S2.**

## Data Availability

All sequencing data generated in this study are available in the GenBank Sequence Read Archive (SRA) under BioProject number PRJNA647983. The genome of *Neurospora crassa* strain ATCC 24698 is available in EnsemblFungi at http://fungi.ensembl.org/Neurospora_crassa/Info/Index at the time of writing. The genome of *Saccharomyces cerevisiae* strain S288C is available in the *Saccharomyces* genome database at http://sgd-archive.yeastgenome.org/sequence/ at the time of writing. The *Sclerotinia sclerotiorum* reference genome is available under BioProject number PRJNA348385 in the National Centre for Biotechnology Information’s GenBank database. The coding DNA sequence of the *S. sclerotiorum* homologue of the *N. crassa* OS1 protein is available from GenBank under accession APA06585.1.

## Abbreviations

HOG: High osmolarity glycerol
MAPK: Mitogen activated protein kinase
CWI: Cell wall integrity

## References

[1] Arima K, Imanaka H, Kousaka M, Fukuta A, Tamura G (1964). Pyrrolnitrin, a new antibiotic substance, produced by Pseudomonas. Agric Biol Chem.

[2] Brandhorst TT, Klein BS (2019). Uncertainty surrounding the mechanism and safety of the post-harvest fungicide fludioxonil. Food Chem Toxicol.

[3] Kilani J, Fillinger S (2016). Phenylpyrroles: 30 years, two molecules and (nearly) no resistance. Front Microbiol.

[4] Corran A, Knauf-Beiter G, Zeun R. Fungicides Acting on Signal Transduction. In: Modern Crop Protection Compounds. 2nd ed. Germany: Wiley-VCH; 2012. p. 715–37.

[5] Hohmann S (2009). Control of high osmolarity signalling in the yeast Saccharomyces cerevisiae. FEBS Lett.

[6] Hohmann S (2002). Osmotic stress signaling and Osmoadaptation in yeasts. Microbiol Mol Biol Rev.

[7] Brewster JL, De Valoir T, Dwyer ND, Winter E, Gustin MC (1993). An osmosensing signal transduction pathway in yeast. Science.

[8] Schumacher MM, Enderlin CS, Selitrennikoff CP (1997). The osmotic-1 locus of Neurospora crassa encodes a putative histidine kinase similar to osmosensors of bacteria and yeast. Curr Microbiol.

[9] Alex LA, Borkovich KA, Simon MI (1996). Hyphal development in Neurospora crassa: involvement of a two-component histidine kinase. Proc Natl Acad Sci U S A.

[10] Miller TK, Renault S, Selitrennikoff CP (2002). Molecular dissection of alleles of the osmotic-1 locus of Neurospora crassa. Fungal Genet Biol.

[11] Zhang Y, Lamm R, Pillonel C, Lam S, Xu JR (2002). Osmoregulation and fungicide resistance: the Neurospora crassa os-2 gene encodes a HOG1 mitogen-activated protein kinase homologue. Appl Environ Microbiol.

[12] Pillonel C, Meyer T (1997). Effect of Phenylpyrroles on glycerol accumulation and protein kinase activity of Neurospora crassa. Pestic Sci.

[13] Zhou F, Hu HY, Song YL, Gao YQ, Liu QL, Song PW (2020). Biological characteristics and molecular mechanism of Fludioxonil resistance in Botrytis cinerea from Henan Province of China. Plant Dis.

[14] Ren W, Shao W, Han X, Zhou M, Chen C (2016). Molecular and biochemical characterization of laboratory and field mutants of botrytis cinerea resistant to fludioxonil. Plant Dis.

[15] Kuang J, Hou YP, Wang JX, Zhou MG (2011). Sensitivity of Sclerotinia sclerotiorum to fludioxonil: in vitro determination of baseline sensitivity and resistance risk. Crop Prot.

[16] Ziogas BN, Markoglou AN, Spyropoulou V (2005). Effect of phenylpyrrole-resistance mutations on ecological fitness of Botrytis cinerea and their genetical basis in Ustilago maydis. Eur J Plant Pathol.

[17] Yoshimi A, Imanishi J, Gafur A, Tanaka C, Tsuda M (2003). Characterization and genetic analysis of laboratory mutants of Cochliobolus heterostrophus resistant to dicarboximide and phenylpyrrole fungicides. J Gen Plant Pathol.

[18] John E, Lopez-Ruiz F, Rybak K, Mousley CJ, Oliver RP, Tan KC (2016). Dissecting the role of histidine kinase and HOG1 mitogen-activated protein kinase signalling in stress tolerance and pathogenicity of parastagonospora nodorum on wheat. Microbiol (United Kingdom).

[19] Bilsland E, Molin C, Swaminathan S, Ramne A, Sunnerhagen P (2004). Rck1 and Rck2 MAPKAP kinases and the HOG pathway are required for oxidative stress resistance. Mol Microbiol.

[20] Lawrence CL, Botting CH, Antrobus R, Coote PJ (2004). Evidence of a new role for the high-Osmolarity glycerol mitogen-activated protein kinase pathway in yeast: regulating adaptation to citric acid stress. Mol Cell Biol.

[21] Mollapour M, Piper PW (2006). Hog1p mitogen-activated protein kinase determines acetic acid resistance in *Saccharomyces cerevisiae*. FEMS Yeast Res.

[22] Aguilera J, Rodriguez-Vargas S, Prieto JA (2005). The HOG MAP kinase pathway is required for the induction of methylglyoxal-responsive genes and determines methylglyoxal resistance in Saccharomyces cerevisiae. Mol Microbiol.

[23] Panadero J, Pallotti C, Rodríguez-Vargas S, Randez-Gil F, Prieto JA (2006). A downshift in temperature activates the high osmolarity glycerol (HOG) pathway, which determines freeze tolerance in Saccharomyces cerevisiae. J Biol Chem.

[24] Thorsen M, Di Y, Tängemo C, Morillas M, Ahmadpour D, Van Der Does C (2006). The MAPK Hog1p modulates Fps1p-dependent arsenite uptake and tolerance in yeast. Mol Biol Cell.

[25] Keller NP, Turner G, Bennett JW (2005). Fungal secondary metabolism — from biochemistry to genomics. Nat Rev Microbiol.

[26] Ochiai N, Tokai T, Nishiuchi T, Takahashi-Ando N, Fujimura M, Kimura M (2007). Involvement of the osmosensor histidine kinase and osmotic stress-activated protein kinases in the regulation of secondary metabolism in Fusarium graminearum. Biochem Biophys Res Commun.

[27] Derbyshire MC, Denton-Giles M (2016). The control of sclerotinia stem rot on oilseed rape ( *Brassica napus* ): current practices and future opportunities. Plant Pathol.

[28] Finn RD, Bateman A, Clements J, Coggill P, Eberhardt RY, Eddy SR (2014). Pfam: the protein families database. Nucleic Acids Res.

[29] Graham-Taylor C, Kamphuis LG, Derbyshire MC (2020). A detailed in silico analysis of secondary metabolite biosynthesis clusters in the genome of the broad host range plant pathogenic fungus Sclerotinia sclerotiorum. BMC Genomics.

[30] Leroch M, Plesken C, Weber RWS, Kauff F, Scalliet G, Hahn M (2013). Gray mold populations in German strawberry fields are resistant to multiple fungicides and dominated by a novel clade closely related to Botrytis cinerea. Appl Environ Microbiol.

[31] Kretschmer M, Leroch M, Mosbach A, Walker A-S, Fillinger S, Mernke D (2009). Fungicide-driven evolution and molecular basis of multidrug resistance in field populations of the Grey Mould fungus Botrytis cinerea. PLoS Pathog.

[32] Briza P, Eckerstorfer M, Breitenbach M (1994). The sporulation-specific enzymes encoded by the DIT1 and DIT2 genes catalyze a two-step reaction leading to a soluble LL-dityrosine-containing precursor of the yeast spore wall. Proc Natl Acad Sci U S A.

[33] Friesen H, Hepworth SR, Segall J (1997). An Ssn6-Tup1-dependent negative regulatory element controls sporulation-specific expression of DIT1 and DIT2 in Saccharomyces cerevisiae. Mol Cell Biol.

[34] Gu Q, Zhang C, Yu F, Yin Y, Shim WB, Ma Z (2015). Protein kinase FgSch9 serves as a mediator of the target of rapamycin and high osmolarity glycerol pathways and regulates multiple stress responses and secondary metabolism in Fusarium graminearum. Environ Microbiol.

[35] Denton-Giles M, Derbyshire MC, Khentry Y, Buchwaldt L, Kamphuis LG (2018). Partial stem resistance in Brassica napus to highly aggressive and genetically diverse Sclerotinia sclerotiorum isolates from Australia. Can J Plant Pathol.

[36] Wickham H (2016). ggplot2: elegant graphics for data analysis.

[37] Team RC (2013). R: a language and environment for statistical computing.

[38] Bolger AM, Lohse M, Usadel B (2014). Trimmomatic: a flexible trimmer for Illumina sequence data. Bioinformatics..

[39] Derbyshire M, Denton-Giles M, Hegedus D, Seifbarghi S, Rollins J, Kan JV (2017). The complete genome sequence of the phytopathogenic fungus Sclerotinia sclerotiorum reveals insights into the genome architecture of broad host range pathogens. Genome Biol Evol.

[40] Turner I, Garimella KV, Iqbal Z, McVean G (2018). Integrating long-range connectivity information into de Bruijn graphs. Bioinformatics..

[41] Cingolani P, Platts A, Wang LL, Coon M, Nguyen T, Wang L (2012). A program for annotating and predicting the effects of single nucleotide polymorphisms, SnpEff. Fly (Austin).

[42] Emms DM, Kelly S (2015). OrthoFinder: solving fundamental biases in whole genome comparisons dramatically improves orthogroup inference accuracy. Genome Biol.

[43] Galagan JE, Calvo SE, Borkovich KA, Selker EU, Read NO, Jaffe D (2003). The genome sequence of the filamentous fungus Neurospora crassa. Nature..

[44] Cherry JM, Adler C, Ball C, Chervitz SA, Dwight SS, Hester ET (1998). SGD: Saccharomyces genome database. Nucleic Acids Res..

[45] Slater G, Birney E (2005). Automated generation of heuristics for biological sequence comparison. BMC Bioinformatics.

[46] Quinlan AR, Hall IM (2010). BEDTools: a flexible suite of utilities for comparing genomic features. Bioinformatics..

[47] Edgar RC (2004). MUSCLE: a multiple sequence alignment method with reduced time and space complexity. BMC Bioinformatics.

[48] Langmead B, Salzberg SL (2012). Fast gapped-read alignment with bowtie 2. Nat Methods.

[49] Robinson MD, McCarthy DJ, Smyth GK (2010). edgeR: a bioconductor package for differential expression analysis of digital gene expression data. Bioinformatics..

[50] Benjamini Y, Hochberg Y (1995). Controlling the false discovery rate: a practical and powerful approach to multiple testing. J R Stat Soc Ser B Methodol.

