# Supplementary figures and images for: Impacts of fludioxonil resistance on global gene expression in the necrotrophic fungal plant pathogen Sclerotinia sclerotiorum
Source: BMC Genomics. 2021 Jan 30;22:91. doi: 10.1186/s12864-021-07402-x (PMC7847169; doi:10.1186/s12864-021-07402-x)

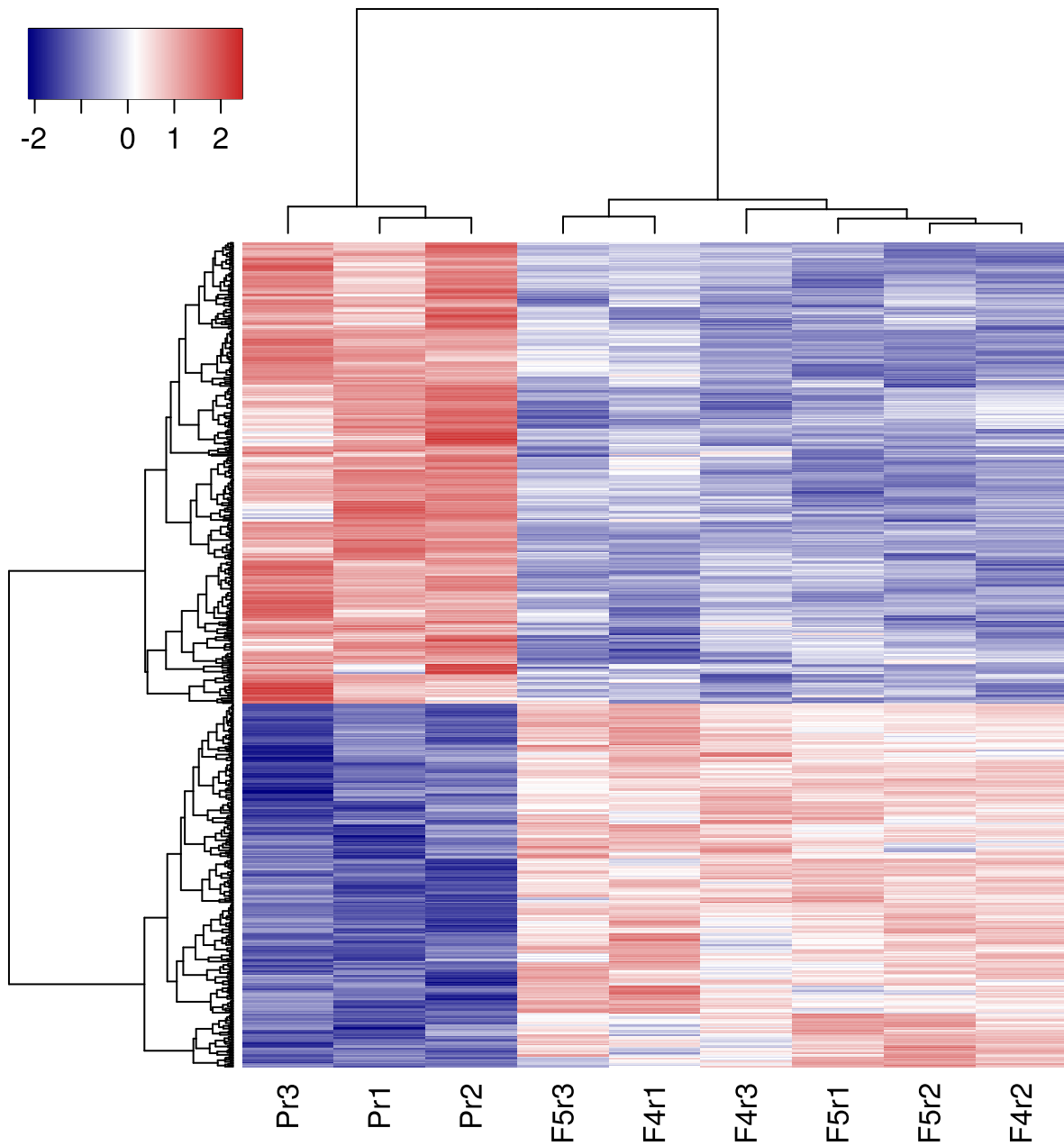

Supplement: Supplementary file 1 — Additional file 1: Figure S1. [file 12864_2021_7402_MOESM1_ESM.pdf]

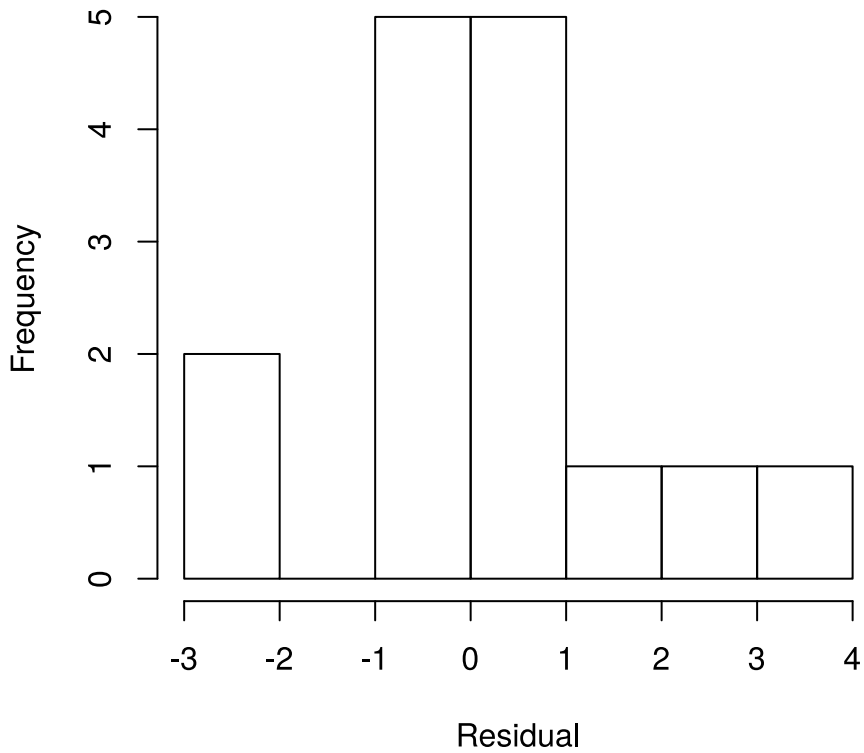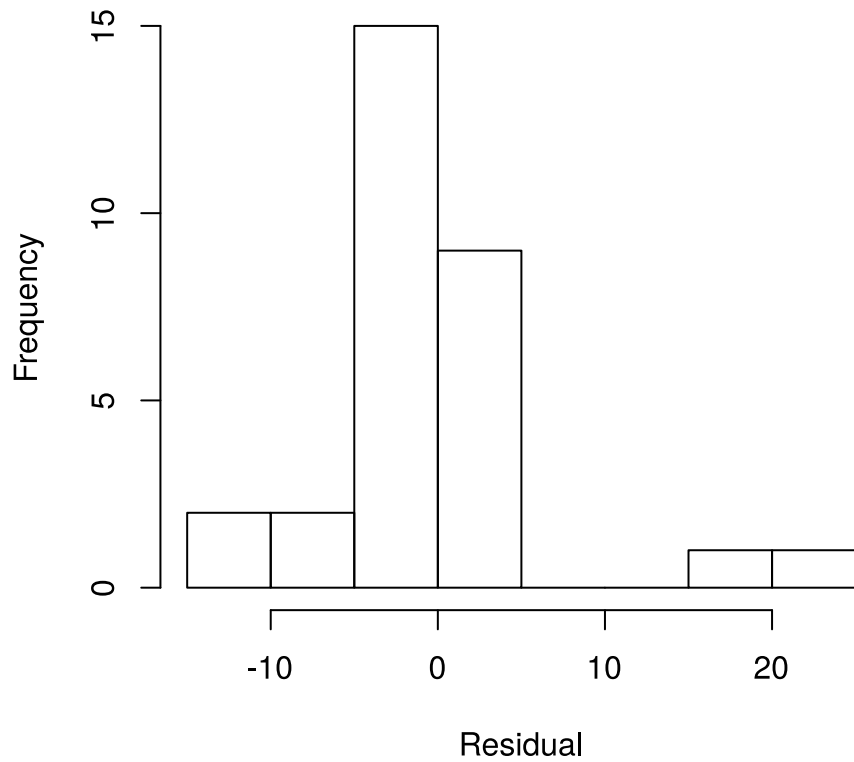

Supplement: Supplementary file 3 — Additional file 3: Figure S2. [file 12864_2021_7402_MOESM3_ESM.pdf]
